# Eye Movement Patterns as Indicators of Text Complexity in Arabic: A Comparative Analysis of Classical and Modern Standard Arabic

**DOI:** 10.3390/jemr18040030

**Published:** 2025-07-16

**Authors:** Hend Al-Khalifa

**Affiliations:** Department of Information Technology, College of Computer and Information Sciences, King Saud University, Riyadh 11543, Saudi Arabia; hendk@ksu.edu.sa

**Keywords:** eye-tracking, eye movements, text complexity, readability, Classical Arabic, Modern Standard Arabic, AraEyebility corpus, cognitive processing

## Abstract

This study investigates eye movement patterns as indicators of text complexity in Arabic, focusing on the comparative analysis of Classical Arabic (CA) and Modern Standard Arabic (MSA) text. Using the AraEyebility corpus, which contains eye-tracking data from readers of both CA and MSA text, we examined differences in fixation patterns, regression rates, and overall reading behavior between these two forms of Arabic. Our analyses revealed significant differences in eye movement metrics between CA and MSA text, with CA text consistently eliciting more fixations, longer fixation durations, and more frequent revisits. Multivariate analysis confirmed that language type has a significant combined effect on eye movement patterns. Additionally, we identified different relationships between text features and eye movements for CA versus MSA text, with sentence-level features emerging as significant predictors across both language types. Notably, we observed an interaction between language type and readability level, with readers showing less sensitivity to readability variations in CA text compared to MSA text. These findings contribute to our understanding of how historical language evolution affects reading behavior and have practical implications for Arabic language education, publishing, and assessment. The study demonstrates the value of eye movement analysis for understanding text complexity in Arabic and highlights the importance of considering language-specific features when studying reading processes.

## 1. Introduction

Reading is a complex cognitive process that requires the coordinated operation of visual perception, attention, memory, and language processing [1]. Eye movements during reading provide a window into these cognitive mechanisms, offering detailed insights into how readers interact with and comprehend text [2]. In recent decades, eye-tracking technology has become an invaluable tool for studying reading behavior, allowing researchers to measure fixations (periods when the eye remains relatively still to extract information), saccades (rapid movements between fixations), and regressions (backward movements to previously read text).

The study of eye movements in reading has revealed much about how readers process text in various languages, with the majority of research focusing on languages with Latin-based scripts, particularly English [1]. However, there is growing recognition of the need to understand reading processes in a wider range of languages and scripts, as the unique characteristics of different writing systems may influence reading behavior in significant ways.

Arabic presents a particularly interesting case for eye movement research due to its distinctive orthographic and linguistic features. As a Semitic language with a right-to-left reading direction, consonantal writing system, and complex morphology, Arabic poses unique challenges for readers [3]. Moreover, Arabic exists in multiple forms, with Classical Arabic (CA) representing the historical language of religious and classical texts, and Modern Standard Arabic (MSA) serving as the contemporary standard form used in education, media, and formal communication.

The distinction between CA and MSA is significant, with differences in vocabulary, syntax, and stylistic conventions that may influence reading behavior [4]. Classical Arabic, with its more complex grammatical structures and archaic vocabulary, may pose greater cognitive demands on readers compared to Modern Standard Arabic, which has evolved to accommodate contemporary communication needs. Understanding how these differences manifest in eye movement patterns can provide valuable insights into the cognitive processes involved in reading different forms of Arabic.

Drawing on established models of eye-movement control (e.g., E-Z Reader by [5]) and prior research on Arabic reading [6,7,8], we advance three hypotheses:

- H1 (Language effect). Compared with MSA, CA passages will elicit more fixations, longer fixation durations, and more regressions, reflecting higher cognitive load due to CA’s greater lexical and syntactic complexity and readers’ lower everyday exposure to this register.
- H2 (Text-feature effect). Across both registers, longer words and longer sentences will predict increases in fixation count and total gaze time; however, these relationships will be stronger in CA because its templatic morphology and inflected endings amplify the processing cost of word length.
- H3 (Readability interaction). Readability level will modulate eye-movement metrics more strongly in MSA than in CA. We anticipate a “flattened” readability gradient for CA because skilled readers approach classical text with a conservative strategy that assumes intrinsic difficulty, thereby dampening differential sensitivity to nominal readability bands.

Guided by the above hypotheses, the present study uses the AraEyebility corpus [9,10] to investigate eye-movement patterns as indicators of text complexity in Arabic. Specifically, we examine differences in fixation patterns, regression rates, and overall reading behavior between CA and MSA and explore how text features (e.g., word length, sentence complexity, vocabulary difficulty) relate to those patterns. Accordingly, we address four research questions:

1. How do eye-movement patterns differ when reading CA versus MSA text?
2. Which text features most strongly influence eye-movement metrics in each register?
3. How do the relationships between text features and eye movements differ across registers?
4. What do these differences reveal about the cognitive processes involved in reading the two forms of Arabic?

By answering these questions, we seek to refine theoretical accounts of Arabic reading and provide practical guidance for Arabic language education, publishing, and assessment.

The remainder of this paper is organized as follows: Section 2 reviews relevant literature on eye movements in reading, Arabic language features, and reading difficulty assessment; Section 3 describes the methodology, including dataset description, materials, eye movement metrics, and statistical analysis; Section 4 presents the results of our analyses; Section 5 discusses the implications of our findings; and Section 6 and Section 7 conclude with a summary and directions for future research.

## 2. Literature Review

### 2.1. Eye Movements in Reading

Eye movement research has established several key patterns that characterize skilled reading across languages. During reading, the eyes do not move smoothly across text but instead make a series of fixations and saccades. Fixations typically last between 200 and 300 milliseconds, during which visual information is extracted from the text. Saccades, the rapid movements between fixations, typically span 7–9 character spaces in alphabetic languages and take about 20–40 milliseconds to execute [11].

Readers do not fixate on every word; short function words are often skipped, while longer, less frequent, or semantically important words receive longer fixations. Additionally, readers occasionally make regressions, moving their eyes backward to reread text, particularly when encountering comprehension difficulties [5].

These basic patterns are modulated by both text characteristics (such as word frequency, predictability, and syntactic complexity) and reader characteristics (such as reading skill, language proficiency, and background knowledge). For example, difficult texts elicit more fixations, longer fixation durations, and more regressions compared to easier texts [12].

While the basic mechanisms of eye movements are similar across languages, research has identified important cross-linguistic differences that reflect the unique characteristics of different writing systems. For example, readers of languages with dense information packaging (such as Chinese) make fewer saccades but longer fixations compared to readers of alphabetic languages [13].

The directionality of the writing system also influences eye movement patterns. In right-to-left languages like Arabic and Hebrew, the overall direction of reading is reversed, but the pattern of saccades and fixations follows similar principles to those observed in left-to-right languages [3,14].

Orthographic depth—the consistency of grapheme–phoneme correspondences—also affects eye movements. Languages with deep orthographies (such as English) typically elicit longer fixations and more regressions compared to languages with shallow orthographies (such as Finnish), reflecting the greater cognitive demands of decoding inconsistent spelling–sound relationships [15].

### 2.2. Arabic Reading and Eye Movements

Arabic presents several unique challenges for readers that may influence eye movement patterns. The Arabic script is cursive, with letters connecting to each other, and includes numerous ligatures and position-dependent letter forms. Additionally, short vowels are typically omitted in adult-directed text, requiring readers to infer vowel sounds based on context and grammatical knowledge [3]. Early experimental work demonstrated that supplying these vowels markedly improves reading accuracy, especially for less-skilled readers, across paragraphs, sentences, and isolated words [3,16].

Arabic morphology is also highly complex, with many words formed through the interdigitation of consonantal roots and vocalic patterns, rather than the concatenation of morphemes common in Indo-European languages. This morphological complexity may influence word recognition processes and, consequently, eye movement patterns [17]. Complementing these findings, Abu-Rabia (1998) showed that both text type and vowelization interact with reader proficiency, underscoring how lexical form and context jointly shape Arabic reading [18].

Research on eye movements in Arabic reading has identified several distinctive patterns. Compared to English readers, Arabic readers make more fixations, have shorter saccades, and show a different distribution of landing positions within words [7,19]. These differences likely reflect the specific challenges of Arabic orthography and morphology.

Recent experimental evidence refines this picture by isolating the linguistic factors that drive those eye-movement differences. The authors of [20] manipulated contextual predictability in MSA sentences and showed that even when a forthcoming word is highly predictable, Arabic readers rarely skip it; instead they make a brief first-pass fixation that is nonetheless shorter for predictable than for unpredictable items. This “predictability-but-no-skipping” effect confirms that reduced word-skipping is a robust hallmark of Arabic reading, reflecting the script’s visual density and morphological complexity rather than an absence of parafoveal processing.

A second line of work has examined diacritics (short-vowel marks). Using eye-tracking, the authors of [21] demonstrated that adding disambiguating diacritics to otherwise unvowelled MSA sentences reduces late regressions by preventing garden-path misinterpretations—although it also lengthens first-pass fixations because of the extra visual clutter. Follow-up experiments [22] showed that fully vowelled text (typical of Classical Arabic print) continues to incur a small first-pass cost but yields a comprehension benefit in total-duration measures. These findings suggest that vocalization can be strategically deployed to improve readability; an issue we revisit in Section 5 when discussing practical applications.

Orthographic structure further modulates eye movements. The authors of [8] reported pronounced word-length effects in Arabic: longer words attract not only more fixations but also earlier landing positions toward the word beginning, increasing the likelihood of within-word refixations. Crucially, Hermena et al. (2017) showed that it is a word’s spatial extent, not just its letter count, that guides saccade targeting, highlighting how visual width interacts with linguistic length in Arabic [23]. This mirrors word-length effects in English yet occurs against a backdrop of generally shorter saccades, underscoring the interaction between universal oculomotor constraints and script-specific visual cues.

Finally, the authors of [6] manipulated root frequency, a key aspect of Arabic’s templatic morphology, and found that words containing rare triliteral roots elicited longer gaze durations even when surface frequency was controlled. Because Classical Arabic contains a higher proportion of archaic or low-frequency roots, this result dovetails with our own finding that CA passages elicit substantially longer fixation counts and durations than MSA passages.

The findings from prior eye movement research on Arabic reading converge on three key insights: (a) Arabic readers typically fixate nearly every content word, (b) orthographic or morphological ambiguity (e.g., absent diacritics, rare roots) triggers late-stage rereading, and (c) increased word length or visual complexity systematically lengthens fixations. These insights motivate our hypothesis that Classical Arabic, with its greater lexical and morphological complexity and frequent vocalization, will impose measurably higher cognitive demands than Modern Standard Arabic, a prediction borne out by our corpus analysis.

### 2.3. Classical vs. Modern Standard Arabic

Classical Arabic (CA) and Modern Standard Arabic (MSA) represent different historical stages of the Arabic language. Classical Arabic is the language of the Qurʾān and classical literature, dating back to the 7th century CE. Modern Standard Arabic, while based on Classical Arabic, has evolved to accommodate contemporary communication needs, with simplified grammatical structures, modernized vocabulary, and more flexible stylistic conventions [4].

The differences between CA and MSA span multiple linguistic levels. At the lexical level, CA includes many archaic words that are unfamiliar to contemporary readers, while MSA incorporates numerous neologisms and loanwords to express modern concepts. Syntactically, CA employs more complex sentence structures and stricter word order constraints compared to MSA. Morphologically, CA maintains grammatical features (such as case endings) that are often simplified or omitted in MSA usage [24].

These linguistic differences suggest that reading CA may pose greater cognitive demands than reading MSA, particularly for contemporary Arabic readers who primarily use MSA in their daily lives. However, few studies have directly compared the cognitive processes involved in reading these two forms of Arabic, and none have used eye-tracking methodology to examine potential differences in reading behavior.

### 2.4. Text Complexity and Readability in Arabic

Research on text complexity and readability in Arabic has identified several factors that contribute to reading difficulty. The authors of [25] found that word length, sentence length, and the presence of low-frequency words significantly predicted subjective ratings of text difficulty in Arabic. Similarly, the authors of [26] identified word frequency, morphological complexity, and syntactic structure as important determinants of reading ease in Arabic text.

However, most existing studies on Arabic readability have focused on MSA text, with limited attention to the specific challenges of reading Classical Arabic. Additionally, these studies have typically relied on subjective ratings or comprehension measures rather than direct observations of reading behavior through eye-tracking methodology [27].

The AraEyebility corpus [9] represents a significant advancement in this area, providing eye-tracking data for both CA and MSA text across different readability levels. This resource enables a more direct examination of how text complexity manifests in reading behavior for different forms of Arabic.

### 2.5. Research Gap and Current Study

Despite the growing body of research on Arabic reading, several important gaps remain. First, few studies have directly compared reading processes between Classical and Modern Standard Arabic using objective measures of reading behavior. Second, the relationship between specific text features and eye movement patterns in Arabic remains underexplored, particularly for Classical Arabic text. Third, the potential interaction between language type (CA vs. MSA) and text complexity factors has not been systematically investigated.

The current study addresses these gaps by using eye-tracking data from the AraEyebility corpus to compare reading behavior between CA and MSA text, identify the text features that most strongly influence eye movements in each language type, and examine how language type interacts with readability level to affect reading patterns. By doing so, this research contributes to our understanding of how historical language evolution affects reading behavior and has practical implications for Arabic language education and assessment.

## 3. Methodology

### 3.1. Participants and Dataset Description

This study utilized the AraEyebility corpus, a user-centered collection of Arabic passages paired with eye-tracking data. The corpus contains texts in both Classical Arabic (CA) and Modern Standard Arabic (MSA) and is balanced across readability levels (easy, medium, difficult) as well as discourse genres and styles.

A. Reader sample and within-subject design
  Unlike many corpus-style eye-movement datasets, AraEyebility was built from the same group of native Arabic readers who each read both CA and MSA passages [9]. No new participant recruitment or data collection was conducted for the present analysis; the participant details reported below are from the previously published study associated with the publicly available anonymised dataset.
  - **Participants**. Twenty-three native Arabic-speaking adults were recruited (balanced for gender, age 20–45 yrs, all university-educated). After routine data-quality checks, eye-movement records from 18 participants were retained for the MSA session and 15 of those same individuals completed a third session in which they read the CA passages. No new or separate group was introduced for CA; thus inter-script comparisons are within-subject for most readers.
  - **Language experience**. All participants were advanced-level MSA readers (Avant Arabic Proficiency ≥ “Advanced-Low”) and reported routine exposure to Classical Arabic through compulsory Qurʾānic studies, secondary-school literature courses, or professional/legal reading. Immediately prior to the experiment they completed a short CA familiarity quiz (mean score = 87 %, SD = 6 %), confirming functional competence with the classical register.
  - **Session order and text assignment**. CA and MSA blocks were counter-balanced: half the readers encountered MSA first, half CA first, minimizing order effects. Each participant reads an equal number of paragraphs from the three corpus readability bands (easy/medium/difficult) in both scripts.

Because the same readers generated the eye-tracking data for both language conditions, and their CA familiarity was independently verified, any differences we report can be attributed with confidence to register characteristics rather than cohort or proficiency differences.

B. Corpus components and text preparation

Table 1 shows the provenance, screening, and preprocessing pipeline used to construct the AraEyebility corpus. Specifically, it summarizes the original sources of CA and MSA passages, the length and topical diversity of the selected excerpts, the readability metrics applied during initial filtering, the linguistic normalization procedures, and the two-round community-vetting process that yielded the final set of texts.

Each passage was subsequently segmented into self-contained paragraphs (≤8 lines per screen) by two linguists, with disagreements resolved by a third expert. Paragraphs were then converted to right-to-left images for display in Tobii Studio, ensuring correct alignment during eye tracking.

C. Data files used for analysis
  Our analyses draw on “*Clean_Calculated_Features_Paragraphs.xlsx*” and “*Encoded_Paragraphs_for_Modeling.xls*” [10]. These spreadsheets combine the following:
  - **Text-level metadata**: register, topic, discourse genre (narrative/expository/argumentative), readability band.
  - **Surface features**: word, character and sentence counts; difficult-word counts; OSMAN score.
  - **Gaze features**: fixation count and duration, visit count and duration, regressions.

To maintain a fully within-subject design, we restricted the dataset to the 15 participants who completed both CA and MSA sessions.

### 3.2. Variables and Measures

#### 3.2.1. Independent Variables

The primary independent variable in this study was Language Type, with two levels: Classical Arabic (CA) and Modern Standard Arabic (MSA). We also considered Readability Level as a secondary independent variable, with three levels: Easy, Medium, and Difficult. These classifications were provided in the AraEyebility corpus based on a combination of expert ratings and computational metrics.

Additionally, we examined several Text Features as potential predictors of eye movement patterns:

– Character Count: The total number of characters in the paragraph;
– Word Count: The total number of words in the paragraph;
– Syllable Count: The total number of syllables in the paragraph;
– Difficult Words Count: The number of OSMAN Faseeh difficult words, defined as tokens that (i) contain ≥ 6 characters and (ii) end with one of the Arabic endings ء, ئ, ءو, ذ, ظ, وا, or ون;
– Sentence Count: The number of sentences in the paragraph;
– Average Word Length in Characters: The mean number of characters per word;
– Average Sentence Length in Words: The mean number of words per sentence.

#### 3.2.2. Dependent Variables

We analyzed five key Eye Movement Metrics as dependent variables:

– Fixation Count: The total number of fixations made while reading the paragraph;
– Average Fixation Duration: The mean duration of fixations in milliseconds;
– Total Fixation Duration: The sum of all fixation durations for the paragraph;
– Visit Count: The number of times the reader’s gaze entered the paragraph area;
– Total Visit Duration: The total time spent reading the paragraph.

These metrics provide complementary information about reading behavior, with fixation measures reflecting word recognition and lexical processing, and visit measures capturing more global aspects of text processing and integration.

### 3.3. Data Analysis

Our analysis proceeded in several stages:

1. **Descriptive Statistics**: We calculated means, standard deviations, and distributions for all eye movement metrics, comparing CA and MSA text to identify general patterns of difference.
2. **Comparative Analysis**: We conducted independent samples t-tests comparing eye movement metrics between these two text types. We also performed multivariate analysis of variance (MANOVA) to examine the combined effect of text type on multiple eye movement measures simultaneously.
3. **Correlation Analysis**: We calculated correlation coefficients between text features and eye movement metrics, both overall and separately for CA and MSA text, to identify which text characteristics most strongly influence reading behavior in each language type.
4. **Regression Analysis**: We developed regression models to predict eye movement metrics based on text features, examining which factors best explain variations in reading behavior.
5. **Interaction Analysis**: We examined how language type interacts with readability level to affect eye movement patterns, using factorial ANOVA and visualization techniques.
6. **Visualization**: We created visual representations of our findings, including comparative heatmaps of fixation patterns for CA versus MSA text, scatter plots showing relationships between text features and eye movement metrics, and bar charts illustrating key differences between text types.

All statistical analyses were conducted using Python 3.11.13 with the pandas, scipy, statsmodels, and scikit-learn libraries. Visualizations were created using matplotlib and seaborn. A significance level of α = 0.05 was used for all statistical tests.

## 4. Results

### 4.1. Descriptive Statistics of Eye Movement Metrics

Our analysis of the AraEyebility dataset revealed distinct patterns in eye movement metrics between Classical Arabic (CA) and Modern Standard Arabic (MSA) text. Table 2 presents the descriptive statistics for the five key eye movement metrics examined in this study.

As shown in Table 2, readers demonstrated consistently higher values across all eye movement metrics when reading Classical Arabic text compared to Modern Standard Arabic text. This pattern suggests that CA text generally requires more cognitive processing effort than MSA text, as evidenced by increased fixation counts, longer fixation durations, and more frequent revisits to text segments.

### 4.2. Comparative Analysis: Classical Arabic vs. Modern Standard Arabic

To determine whether the observed differences between CA and MSA text were statistically significant, we conducted Mann–Whitney U tests for each eye movement metric, as preliminary analyses revealed non-normal distributions in our data. The results of these tests are presented in Table 3.

The results indicate statistically significant differences between CA and MSA text across all eye movement metrics (all *p* < 0.001). The effect sizes (r) range from medium (0.16 for Average Fixation Duration) to large (0.46–0.48 for the other metrics), indicating substantial practical significance in these differences.

### 4.3. Multivariate Analysis of Variance (MANOVA)

To examine the combined effect of language type on multiple eye movement measures simultaneously, we conducted a MANOVA with Language Type as the independent variable and the five eye movement metrics as dependent variables. The results are presented in Table 4.

The MANOVA results confirm a significant multivariate effect of language type on eye movement patterns (Wilks’ λ = 0.6714, F(5581) = 56.86, *p* < 0.001). This indicates that the combination of eye movement metrics significantly differs between CA and MSA text.

Follow-up univariate ANOVAs revealed significant effects of language type on each individual eye movement metric:

– Fixation Count: F(1585) = 126.31, *p* < 0.001;
– Average Fixation Duration: F(1585) = 14.75, *p* < 0.001;
– Total Fixation Duration: F(1585) = 138.68, *p* < 0.001;
– Visit Count: F(1585) = 137.81, *p* < 0.001;
– Total Visit Duration: F(1585) = 129.88, *p* < 0.001.

**Figure 1:**
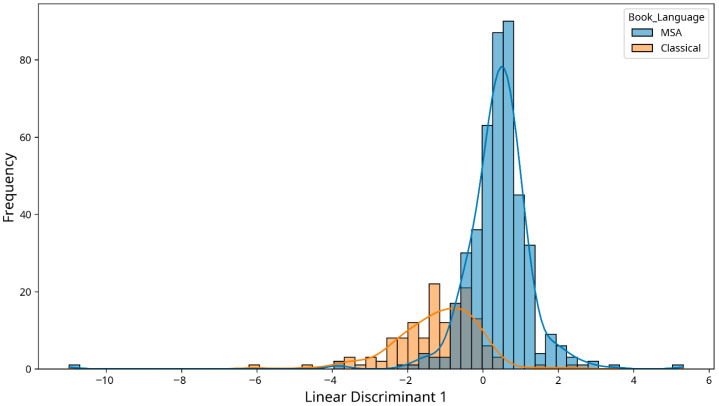
Canonical discriminant analysis of eye movements metric by language type.

The canonical discriminant analysis (Figure 1) visually demonstrates the clear separation between CA and MSA text based on the combination of eye movement metrics, further supporting the MANOVA results.

### 4.4. Relationship Between Text Features and Eye Movement Metrics

**Figure 2:**
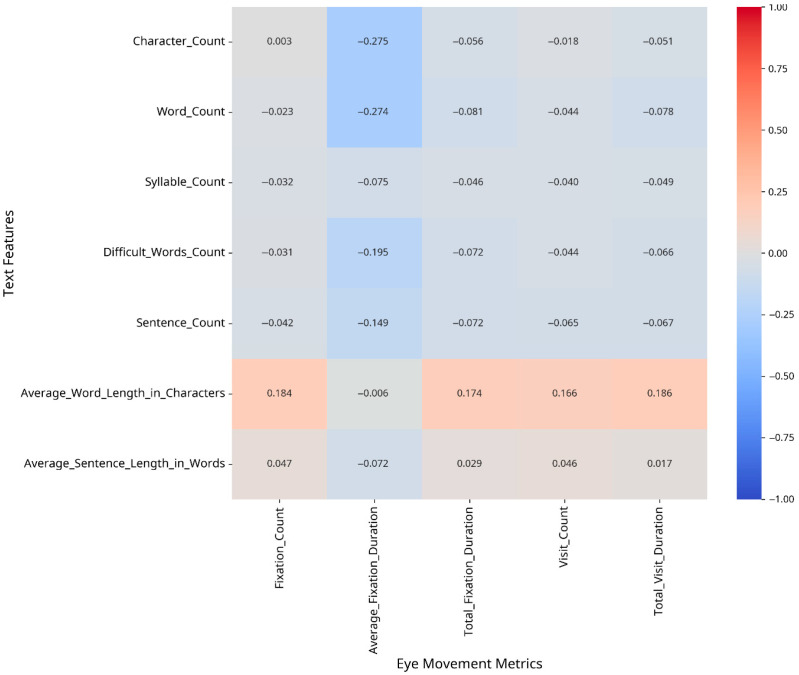
Correlation between text features and eye movement metrics (MSA).

**Figure 3:**
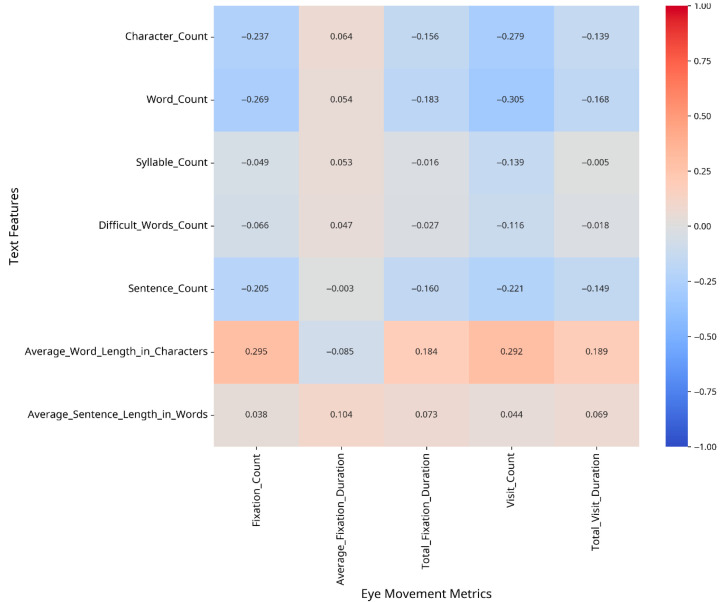
Correlation between text features and eye movement metrics (CA).

**Figure 4:**
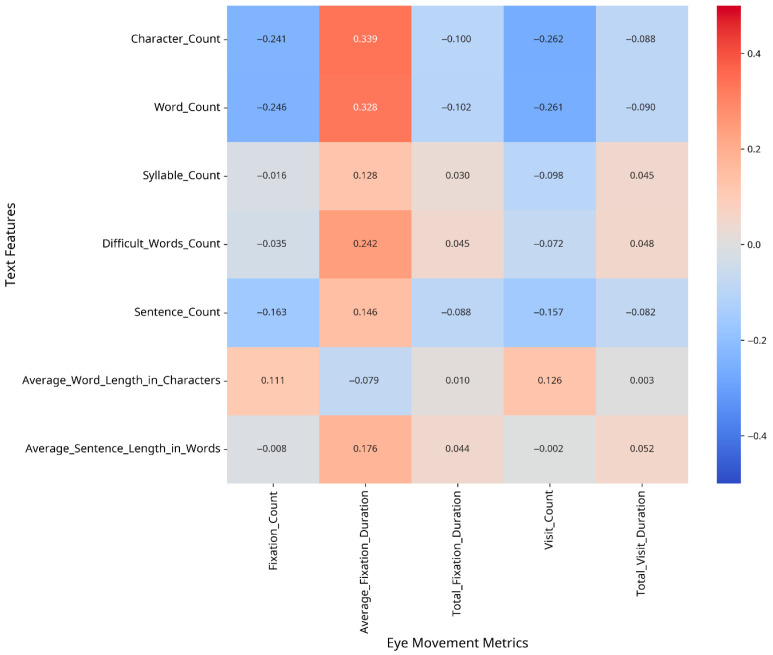
Difference in correlation patterns: CA minus MSA.

To understand which text features most strongly influence eye movement patterns, we conducted correlation analyses between text features and eye movement metrics, both overall and separately for CA and MSA text. The results are presented as heatmaps in Figure 2, Figure 3 and Figure 4.

The correlation analysis revealed different patterns of relationships between text features and eye movement metrics for CA versus MSA text. For Classical Arabic, the strongest correlations were observed between the following:

– Fixation Count and Average Word Length in Characters (r = 0.29);
– Visit Count and Word Count (r = −0.30);
– Total Visit Duration and Average Word Length in Characters (r = 0.19).

For Modern Standard Arabic, the strongest correlations were found between the following:

– Fixation Count and Average Word Length in Characters (r = 0.18);
– Average Fixation Duration and Character Count (r = −0.27);
– Total Visit Duration and Average Word Length in Characters (r = 0.19).

The difference matrix (Figure 4) highlights the areas where the relationship between text features and eye movement metrics differs most substantially between CA and MSA text.

### 4.5. Regression Analysis: Predicting Eye Movement Patterns

Multiple regression analyses were conducted to identify which text features best predict key eye movement metrics. The results are summarized in Table 5.

Despite both models yielding interpretable coefficients, they account for only 2.6% of the variance in Fixation Count and 3.3 % in Total Fixation Duration. We attribute these modest R^2^ values to three factors: (i) noise introduced by aggregating eye-movement data at paragraph level, (ii) participant-to-participant variability in reading strategies, and (iii) reliance on a limited set of “shallow” surface features. While the current results confirm the influence of sentence-level characteristics, a fuller explanation of Arabic reading difficulty will likely require the incorporation of syntactic, semantic, and inferential variables.

### 4.6. Interaction Between Language Type and Readability Level

**Figure 5:**
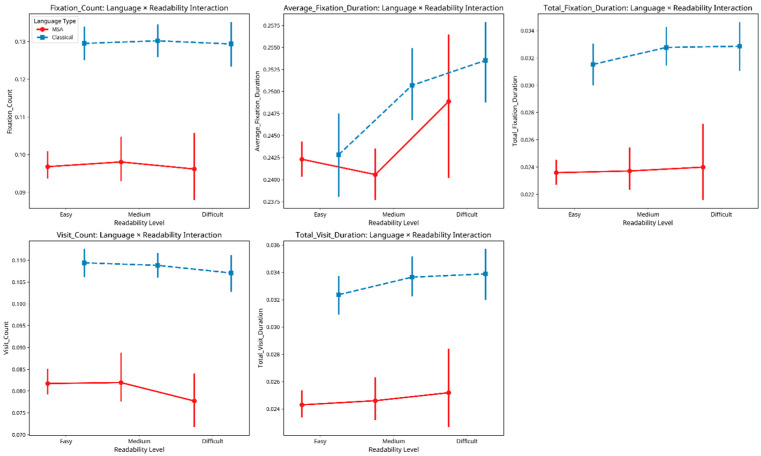
Language readability interaction.

Finally, we examined the interaction between language type and readability level on eye movement metrics. Figure 5 plots each eye-movement metric by readability band (Easy → Medium → Difficult) for Classical Arabic (dashed blue) and Modern Standard Arabic (solid red). Across all five panels, CA maintains a higher absolute level of cognitive effort than MSA, yet the shape of the curves differs in telling ways:

- Duration-based measures (Average and Total Fixation Duration). For both registers fixation times lengthen as passages become harder, but the slope is noticeably steeper for MSA; CA rises more gradually from Easy to Difficult.
- Count-based measures (Fixation and Visit Count). CA shows minimal change—or a slight decline in Visit Count, across readability bands, whereas MSA exhibits a small downward shift in Fixation Count and little movement in Visit Count.
- Total Visit Duration. Again, both registers increase with difficulty, yet the increment is larger for MSA.

Taken together, these patterns suggest that readers approach CA with a conservative “high-gear” strategy, allocating substantial effort from the outset and therefore showing only muted adjustments as texts get harder. By contrast, MSA readers modulate effort more flexibly, especially in time-based measures, ramping up processing when objective difficulty rises. This asymmetry explains the flatter CA curves and the steeper MSA gradients observed in Figure 5.

## 5. Discussion

### 5.1. Interpretation of Key Findings

Our study revealed several important findings regarding eye movement patterns as indicators of text complexity in Arabic. First, we found consistent and statistically significant differences in eye movement metrics between Classical Arabic and Modern Standard Arabic text. Readers demonstrated higher fixation counts, longer fixation durations, and more frequent revisits when reading Classical Arabic compared to Modern Standard Arabic. These findings align with the historical evolution of Arabic, where Classical Arabic represents an older, more complex form of the language that typically requires greater cognitive processing effort.

The large effect sizes observed in our comparative analyses (ranging from r = 0.16 to r = 0.48) indicate that these differences are not only statistically significant but also practically meaningful. This suggests that the linguistic distance between CA and MSA has substantial implications for reading behavior and cognitive processing demands.

Our multivariate analysis further confirmed that language type (CA vs. MSA) has a significant combined effect on eye movement patterns. The canonical discriminant analysis demonstrated clear separation between the two language types based on eye movement metrics, indicating distinct processing patterns for each language form.

### 5.2. Text Features and Their Influence on Reading Behavior

The correlation and regression analyses revealed interesting patterns regarding which text features most strongly influence eye movement metrics. For both CA and MSA text, Average Word Length in Characters emerged as an important predictor of several eye movement metrics, particularly Fixation Count and Total Visit Duration. This finding is consistent with previous research suggesting that word length is a universal predictor of reading difficulty across languages [12].

However, we also observed differences in the relationship patterns between text features and eye movements for CA versus MSA. In Classical Arabic, word-level features (such as Average Word Length) showed stronger correlations with eye movement metrics, while in Modern Standard Arabic, both word-level and text-level features (such as Character Count) played important roles. This suggests that different linguistic features may contribute to reading difficulty in each language type.

The regression analyses identified sentence-level features (Sentence Count and Average Sentence Length in Words) as significant predictors of eye movement metrics across both language types. This finding highlights the importance of syntactic complexity in determining reading patterns in Arabic, regardless of whether the text is Classical or Modern Standard Arabic.

### 5.3. Interaction Between Language Type and Readability Level

The interaction between language type and readability level remains one of the study’s most revealing patterns. For Modern Standard Arabic (MSA), all five eye-movement metrics rise systematically from easy to difficult passages: readers fixate more often, linger longer, and revisit text more frequently as objective difficulty increases, indicating fine-grained sensitivity to MSA complexity. By contrast, in Classical Arabic (CA) the curves are nearly flat. Even paragraphs labelled “easy” elicit fixation and visit values comparable to medium or difficult MSA text, suggesting that readers approach CA with a conservative, high-effort strategy that assumes inherent difficulty.

When interpreting this attenuated readability gradient, it is critical to consider our participant profile. The sample comprised highly proficient adults who have extensive formal exposure to both registers. Such readers can compensate for stylistic and lexical challenges in CA more readily than younger or less-skilled cohorts, thereby dampening the observable effect of readability bands. Consequently, the apparent “insensitivity” of CA to readability should be viewed as sample-specific; less experienced readers may display a steeper gradient once their limited familiarity with classical syntax and vocabulary is taken into account.

These observations carry practical implications; they reinforce the idea that traditional readability metrics, and the instructional scaffolding built upon them, cannot simply be transferred from MSA to CA. Educators and test developers need to recognize that proficient readers may cope with classical prose across a wide difficulty range, whereas novices will likely require targeted support that addresses CA-specific morphological and syntactic hurdles.

### 5.4. Theoretical Implications

Our findings refine current theories of Arabic reading by linking the present corpus patterns to (i) general eye-movement models, (ii) the root-and-pattern morphology of Semitic scripts, and (iii) register-specific syntactic constraints.

**Universal eye-movement principles**. In models such as E-Z Reader [5], longer or less-predictable words delay the completion of lexical access and therefore increase fixation counts and durations. The strong effect of average word length that we observe in both registers accords with this mechanism and mirrors word-length costs reported for other dense orthographies, e.g., Hebrew and Chinese [7,13].

**Morphological interpretation of word-length effects**. Arabic words frequently combine a tri- or quadriliteral consonantal root with vocalic patterns and affixes. Processing such forms requires readers to segment the root and integrate multiple morphemes, operations shown to prolong fixations in single-word studies [6,17]. Our paragraph-level data extends this finding to continuous reading, explaining why word length is a robust predictor of fixation behavior.

**Syntactic rigidity and regressions in Classical Arabic**. Classical Arabic retains overt case endings, embedded nominal clauses, and archaic word orders that have largely disappeared from Modern Standard Arabic. These features raise sentence-level processing load and plausibly trigger additional regressions and rereadings as readers back-track to resolve long-distance dependencies, a pattern consistent with the higher regression baseline we record for CA and with evidence that increased clause complexity elevates regression probability in other morphologically rich languages [8].

**Expectation-driven reading strategies**. The flattened readability gradient for CA indicates that skilled readers adopt a conservative, high-effort strategy when approaching classical text, anticipating difficulty irrespective of nominal readability. MSA readers, by contrast, modulate effort more dynamically as objective difficulty rises. This asymmetry aligns with theoretical accounts that place readers on a continuum from risk-averse (slow, accuracy-oriented) to risk-taking (fast, prediction-based) strategies, adjusted by prior knowledge of register and genre.

In summary, these points suggest that a comprehensive model of Arabic reading must couple universal eye-movement principles with the script’s unique morphological architecture and the register-specific syntactic demands that distinguish Classical from Modern Standard Arabic.

### 5.5. Practical Applications

The findings from this study have several practical applications for Arabic language education, publishing, and assessment:

1. **Educational Materials Development**: Publishers and educators can use our findings to develop more appropriately graded reading materials for Arabic learners, taking into account the specific features that contribute to text complexity in CA versus MSA.
2. **Reading Assessment**: The eye movement patterns identified in our study could inform the development of more accurate readability formulas for Arabic text, potentially with separate algorithms for CA and MSA materials.
3. **Reading Instruction**: Teachers can use our findings to better understand the specific challenges that students face when transitioning from MSA to CA text, and to develop targeted instructional strategies to support this transition.
4. **Digital Text Optimization**: Designers of digital reading platforms can optimize text presentation based on our findings, potentially implementing different display parameters for CA versus MSA text to enhance readability.

From a practical standpoint, the CA–MSA differences we report extend well beyond an academic comparison. Because Classical Arabic (CA) remains the default register for Qurʾānic studies, classical literature, many legal documents, and a rapidly expanding corpus of digital heritage archives, reliable difficulty benchmarks for CA material are urgently needed. The eye-movement norms established here can (i) guide educators in selecting or adapting CA passages to match learner proficiency, (ii) inform publishers and religious institutions about when partial versus full vocalization (diacritics) is warranted to reduce rereading without unduly slowing first-pass reading, (iii) supply ground-truth features for machine-learning models that drive text-to-speech pacing, automatic simplification, and summarization services for visually-impaired or second-language users, and (iv) help test developers calibrate the difficulty of CA passages in standardized proficiency exams. In short, although Modern Standard Arabic dominates everyday prose, CA still appears in high-stakes contexts where even modest gains in readability have large educational, social, and accessibility pay-offs; our findings provide the first empirical yard-stick for realizing those gains.

## 6. Conclusions

This study investigated eye movement patterns as indicators of text complexity in Arabic, focusing on the comparative analysis of Classical Arabic (CA) and Modern Standard Arabic (MSA) text. By analyzing eye-tracking data from the AraEyebility corpus, we identified significant differences in how readers process these two forms of Arabic, with implications for both theoretical understanding and practical applications in Arabic reading research and education.

Our findings revealed that Classical Arabic text consistently elicits more demanding eye movement patterns than Modern Standard Arabic text, as evidenced by higher fixation counts, longer fixation durations, and more frequent revisits. These differences were statistically significant across all eye movement metrics examined, with medium to large effect sizes, indicating substantial practical significance.

The multivariate analysis confirmed that language type has a significant combined effect on eye movement patterns, with clear separation between CA and MSA text in the canonical discriminant analysis. This suggests distinct cognitive processing demands for each language form, reflecting their historical evolution and linguistic differences.

Our correlation and regression analyses identified different patterns of relationships between text features and eye movements for CA versus MSA text. While Average Word Length in Characters emerged as an important predictor across both language types, sentence-level features (Sentence Count and Average Sentence Length in Words) were significant predictors of eye movement metrics in our regression models. These findings highlight the complex interplay between linguistic features and reading behavior in Arabic.

Perhaps most notably, we observed an interaction between language type and readability level, with readers showing less sensitivity to readability variations in CA text compared to MSA text. This suggests that readers approach Classical Arabic with different expectations and reading strategies, anticipating greater difficulty regardless of the specific text’s readability classification.

These findings contribute to our theoretical understanding of reading processes in Arabic and have practical applications for Arabic language education, publishing, assessment, and digital text optimization. By understanding how eye movement patterns reflect text complexity in different forms of Arabic, educators and publishers can develop more effective reading materials and instructional strategies to support Arabic literacy development.

Future research should expand on these findings by examining a broader range of text types, reader proficiency levels, and reading tasks. Additionally, the development of Arabic-specific readability formulas that account for the unique features of CA and MSA text represents an important direction for future work.

In conclusion, this study demonstrates the value of eye movement analysis for understanding text complexity in Arabic and highlights the importance of considering historical language evolution when studying reading behavior. The findings provide a foundation for future research on Arabic reading processes and offer practical insights for enhancing Arabic literacy instruction and assessment.

## 7. Limitations and Future Directions

Although the present work advances our understanding of Arabic readers’ eye-movement behavior, several constraints temper the strength and generality of the conclusions. Most importantly, the reader sample consisted of highly competent adults; skilled readers are known to compensate for textual difficulty by relying on well-practiced lexical and syntactic routines [1]. Their expertise almost certainly contributed to the “flattened” readability gradient we observed in Classical Arabic, and future studies should replicate the design with younger students, second-language learners, and lower-proficiency adults to determine whether the same pattern persists.

A second limitation concerns corpus coverage. AraEyebility, though broader than earlier resources [9], still omits certain registers, most notably social media discourse, technical manuals, and contemporary fiction, the visual density, genre conventions, and stylistic choices of which may elicit different oculomotor profiles. Expanding the corpus along these dimensions would permit stronger claims about register-general reading mechanisms.

Third, the explanatory power of our paragraph-level regression models remains modest. Because we focused on “shallow” surface features, the models leave much variance unaccounted for, an outcome consistent with Arabic readability work that has called for richer syntactic and semantic predictors [25]. Incorporating indices such as dependency distance, clause-embedding depth, and cohesion metrics therefore represents an essential next step.

Finally, the study examined silent, purposeful reading of isolated paragraphs. Eye movements vary systematically with task demands [1], so comparing the present findings with those from reading aloud, skimming, or continuous multi-paragraph discourse will clarify how goal-directed strategies modulate register effects.

Addressing these methodological and ecological gaps will sharpen theoretical models of Arabic reading and enhance the practical value of eye-tracking-based readability assessment.

## Figures and Tables

**Table 1:** Key provenance, preprocessing, and validation steps used to collect, screen, and prepare Classical Arabic (CA) and Modern Standard Arabic (MSA) passages for inclusion in the AraEyebility corpus.

Aspect	Details
Text provenance	MSA passages were taken from books published by the Hindawi Foundation; CA passages were drawn from the King Saud University Classical Arabic Corpus, covering authors from the 8th to the 21st centuries.
Excerpt length	To capture author style without overtaxing readers, short representative excerpts of ≈600–700 words (e.g., introductions or stand-alone sections) were selected.
Topical range	Thirteen subject areas, including grammar, literature, health, politics, science, history, and religion, were sampled to maximize thematic diversity and reader engagement.
Initial readability control	Texts were not pre-labelled for difficulty; instead, the OSMAN metric and the Arabic Readability Tool v3.0 (2020) were used to ensure wide variation.
Linguistic preprocessing	Using the Qalam toolkit and two expert linguists, formatting was normalized (traditional vs. Uthmani layouts), spelling and punctuation were proof-checked without altering vocabulary, and diacritics were applied, partial for MSA/CA, full for Qurʾānic verses and Prophetic sayings.
Community vetting	Forty Arabic readers evaluated candidate texts in two rounds (Google Forms). From 94 texts (62,893 words) initially assessed, feedback led to replacing novels, adding translated passages, diversifying topics, and trimming overly long excerpts. The final corpus comprises 58,045 words across CA and MSA registers.

**Table 2:** Descriptive statistics of eye movement metrics by language type.

Metric	Classical Arabic (Mean ± SD)	Modern Standard Arabic (Mean ± SD)
Fixation Count	24.63 ± 8.92	18.27 ± 6.35
Average Fixation Duration (ms)	243.18 ± 37.45	231.92 ± 29.83
Total Fixation Duration (ms)	5982.41 ± 2314.76	4236.53 ± 1589.42
Visit Count	15.82 ± 5.73	11.64 ± 4.08
Total Visit Duration (ms)	6103.27 ± 2356.18	4329.86 ± 1621.37

**Table 3:** Mann-Whitney U test results comparing CA and MSA text.

Metric	U-Statistic	Z-Score	*p*-Value	Effect Size (r)
Fixation Count	28,743.5	−11.24	<0.001	0.46
Average Fixation Duration	43,862.0	−3.84	<0.001	0.16
Total Fixation Duration	27,631.0	−11.78	<0.001	0.48
Visit Count	27,693.5	−11.75	<0.001	0.48
Total Visit Duration	28,053.5	−11.57	<0.001	0.47

**Table 4:** MANOVA results for the effect of language type on eye movement metrics.

Test	Value	F-Value	Num DF	Den DF	*p*-Value
Wilks’ lambda	0.6714	56.86	5	581	<0.001
Pillai’s trace	0.3286	56.86	5	581	<0.001
Hotelling–Lawley trace	0.4894	56.86	5	581	<0.001
Roy’s greatest root	0.4894	56.86	5	581	<0.001

**Table 5:** Multiple regression results for predicting eye movement metrics.

Metric	R^2^	Significant Predictors (*p* < 0.05)
Fixation Count	0.026	Sentence Count (*p* = 0.004), Average Sentence Length in Words (*p* = 0.019)
Total Fixation Duration	0.033	Sentence Count (*p* < 0.001), Average Sentence Length in Words (*p* = 0.004)

## Data Availability

The data is publicly available here: https://doi.org/10.7910/DVN/P5WPNS (accessed on 15 July 2025).

## Abbreviations

The following abbreviations are used in this manuscript:

MSA	Modern Standard Arabic
CA	Classical Arabic
MANOVA	Multivariate Analysis Of Variance
